# A Randomized, Double-Blind, Single-Dose Study Comparing the Biosimilarity of HOT-1010 With Bevacizumab (Avastin®) in Chinese Healthy Male Subjects

**DOI:** 10.3389/fphar.2021.694375

**Published:** 2021-06-17

**Authors:** Kai Huang, Linling Que, Ying Ding, Nannan Chu, Zhenzhong Qian, Yunfei Shi, Wei Qin, Zhenni Li, Yuanxin Chen, Xianghong Gu, Jiakun Wang, Lin Zhang, Jisheng Zhang, Xiangyang Zhu, Yongmin Yang, Yuan Tang, Qing He

**Affiliations:** ^1^ Drug Clinical Trial Institution, Affiliated Wuxi People’s Hospital of Nanjing Medical University, Wuxi, China; ^2^ Shanghai Huaota Biopharmaceutical Co., Ltd., Shanghai, China

**Keywords:** biosimilarity, pharmacokinetics, bevacizumab, Immunogenicity, Avastin®, HOT-1010

## Abstract

**Objective:** This study was conducted to compare the pharmacokinetics, safety and immunogenicity of HOT-1010 with bevacizumab (Avastin®) in Chinese healthy male subjects.

**Methods:** A single-center, randomized, double-blind, single-dose, parallel trial was performed in 84 Chinese healthy male subjects who randomly (1:1) received a single intravenous infusion of 1 mg/kg HOT-1010 or Avastin® for 90 min and followed up for 85 days. Serum concentrations of bevacizumab were analyzed by enzyme-linked immunosorbent assay. Primary pharmacokinetic parameters, C_max_, AUC_0-t_ and AUC_0-∞,_ were calculated and evaluated the bioequivalence between HOT-1010 and Avastin®, the safety and immunogenicity of investigational drugs were also assessed.

**Results:** A total of 82 subjects completed the study. The 90% Confidence Intervals for geometric mean ratios of C_max_, AUC_0-t_ and AUC_0-∞_ were 91.81–103.64%, 85.19–95.39% and 85.04–95.36%, which were all within the bioequivalence margin. Treatment-emergent adverse events were reported in 27 (65.9%) subjects in HOT-1010 group and 23 (56.1%) subjects in Avastin® group. Most TEAEs were mild or moderate. No TEAEs, Serious Adverse Events or deaths leading to discontinuation was reported. Subjects were all tested negative for Anti-drug Antibody.

**Conclusion:** HOT-1010 exhibited the similar pharmacokinetics, safety and immunogenicity profiles of bevacizumab (Avastin®) in Chinese healthy male subjects.

**Clinical Trial Registration:** http://www.chinadrugtrials.org.cn/index.html, CTR20181610.

## Introduction

Bevacizumab (Avastin®) is a recombinant humanized monoclonal IgG1 antibody that binds to the human vascular endothelial growth factor (VEGF) which is essential for both the normal cell and tumor angiogenesis (Li and Kroetz, 2018). Bevacizumab could prevent the activation of VEGF tyrosine kinase receptors (VEGFR1 and VEGFR2) by neutralizing VEGF. The anti-tumor effect of bevacizumab is primarily attributed to the inhibition of VEGFR2-mediated angiogenesis, thereby slowing the growth of new blood vessels and effectively cutting off the supplement of oxygen and nutrients to the tumor (Ferrara et al., 2004; Garcia et al., 2020; Singh et al., 2020). Additionally, inhibition of the VEGF signaling pathway also improves cytotoxic drugs delivery by lowering tumor interstitial fluid pressure and reducing the number of non-functional tumor blood vessels (Garcia et al., 2020).

As the first VEGF targeting drug, bevacizumab has been officially approved by the United States Food and Drug Administration (USFDA) for cancer therapy, such as metastatic colorectal cancer (mCRC), non-small cell lung cancer (NSCLC), renal cell carcinoma (RCC), glioblastoma multiforme (GBM), cervical cancer (CC), and ovarian cancer (OC) (Ferrara and Adamis, 2016). It was also used to treat metastatic breast cancer (mBC) in the European Union and other non-U.S. countries (Li and Kroetz, 2018). Bevacizumab combined with the standard chemotherapy has been confirmed to prolong overall survival and progression-free survival, and improve the overall response rate in various solid tumor indications (DE Marinis et al., 2017; Wu et al., 2017), which offers a novel treatment option and new hope for cancer patients.

The biosimilar is a biological product that is highly similar, but not identical, to the licensed biological product. USFDA, European Medicines Agency (EMA) and the National Medical Products Administration (NMPA) have all emphasized the stepwise strategy for the development of biosimilar by pharmaceutical, preclinical and clinical comparative studies FDA US, 2015; EMA, 2014; FDA CHINA, 2015). The analytical and bio-functional similarity should be firstly demonstrated between biosimilar and originator product, and then the similarity of PK characteristics was evaluated by pharmacokinetic (PK) study in healthy subjects and the efficacy was demonstrated by pharmacodynamic (PD) study in sensitive patients. More importantly, the safety and immunogenicity should be considered seriously throughout the development (FDA US, 2015; EMA, 2014; FDA CHINA, 2015; WHO, 2010).

HOT-1010 is a potential biosimilar of bevacizumab developed by Shanghai Huaota Biopharmaceutical Co., Ltd. Nowadays, all the nonclinical studies of HOT-1010 have been completed, and the results confirmed that HOT-1010 had the highly similar physiochemical properties, bioactivities and PK profiles to bevacizumab (data not shown). Based on these systematic research results, the clinical trial of HOT-1010 has been approved by NMPA (approval NO.: 2018L02957). PK study in humans is an initial and vital step to evaluate the difference in PK profiles between biosimilar and reference product. In our study, the primary objective was to compare the biosimilarity of HOT-1010 with Avastin® in Chinese healthy male subjects. Meanwhile, the safety and immunogenicity of HOT-1010 vs. Avastin® were also evaluated.

## Methods

### Study Population

Chinese healthy male subjects aged 18–60 years, with a body mass index of 18.0–26.0 kg/m^2^ and body weight of 50.0–80.0 kg, were enrolled in this study. All subjects were evaluated to be healthy by vital signs, physical examination, medical history (such as malignancy, respiratory disease, nerve system disease, digestive disease, kidney disease, family history, hemorrhagic and thromboembolic event), laboratory tests (blood chemistry, hematology, coagulation and urinalysis), virological examinations (HIV antibody, hepatitis B antigen, hepatitis C antibody and *Treponema pallidum* antibody), chest X-ray, abdominal ultrasound, electrocardiograph (ECG), urine nicotine test, alcohol breath analysis and urine drug screening test within 2 weeks before the study. In addition, subjects were excluded if they were allergic to any biologic agents, took any medicine (including vitamins, over-the-counter products, herbal products or dietary supplements) within the preceding 30 days, took part in any clinical trial within the past 3 months or received any monoclonal antibodies treatment within 12 months prior to screening.

The screening was performed 14 days before dosing. Eventually, 84 male subjects fulfilled all the inclusion criteria and were admitted to the phase I center through rigorous screenings. They were randomized into HOT-1010 group and Avastin® group, which consisting of 42 subjects in each group. All subjects fasted for at least 10 h prior to dosing and received an intravenous infusion of 1 mg/kg HOT-1010 (Shanghai Huaota Biopharmaceutical Co., Ltd., Shanghai, China; Batch number: 20180101) or Avastin® (Roche Diagnostics GmbH, Penzberg, Germany, EU; Batch number: H0190B05) for 90 min with 100 ml 0.9% sodium chloride injection.

### Study Design

A single-center, randomized, double-blind, parallel trial was conducted to compare the pharmacokinetics, safety and immunogenicity of HOT-1010 with Avastin® in Chinese healthy male subjects.

This clinical trial was performed at the phase I center of Wuxi people’s hospital between June 2019 and December 2019 (Chinese Clinical Trial Registry, Registration No. CTR20181,610; http://www.chinadrugtrials.org.cn/index.html). The final protocol, informed consent documentation and other files were all reviewed and approved by the independent Ethics Committee of Wuxi people’s hospital (NO: 2019LLPJ-I-05). This study was carried out according to good clinical practice (GCP) and the principle of the Declaration of Helsinki. All subjects were informed of the objective, content and risks of the study and voluntarily signed the written informed consent form before enrollment.

### Pharmacokinetic Analysis

5.0 ml blood samples for PK analysis were collected in vacuum tubes containing separation gel at 0 h (pre-dose), 0.75, 1.5 (end of infusion), 3.5, 5.5, 8, 12, 24, 48 and 96 h, then on Days 8 (168 h), 15 (336 h), 22 (504 h), 29 (672 h), 36 (840 h), 43 (1,008 h), 57 (1,344 h), 71 (1,680 h) and 85 (2016 h) after the start of infusion. All samples were gently mixed 5–10 times, and then stood for at least 30 min at room temperature. After that, samples were centrifuged at 1,500 g for 15 min at 4°C. Serum samples were collected and stored at −80°C until analyzed.

The serum concentrations of the bevacizumab biosimilar (HOT-10101) and bevacizumab were analyzed by enzyme-linked immunosorbent assay (ELISA). The calibration curve was validated over the concentration range of 200.00–12,800.00 ng/ml with the lower limit of quantification (LLOQ) of 200.00 ng/ml. The intra-day and inter-day precision of quality control (QC) samples did not exceed 7.4%, while the accuracy was within ±11.8%. The drug concentrations in serum samples which exceed the upper limit of quantification (ULOQ) should be diluted, and 32-fold dilutions did not affect the accuracy and precision. Additionally, QC samples at two concentrations (LQC: 600.00 ng/ml and HQC: 10,000 ng/ml) were stable at ambient temperature for 48 h, 2–8°C for 48 h, −20°C for 47 days and −80°C for 47 days, respectively.

### Safety Evaluation

Safety was assessed by clinical observation and spontaneous reporting of adverse events (AEs) during the study. Clinical laboratory tests (blood chemistry, hematology, coagulation and urinalysis), 12-lead ECG, physical examination and vital signs were also assessed at the end of the study. All AEs were coded according to the Medical Dictionary for Regulatory Activities (MedDRA®) (version 20.0), and the seriousness and severity of AEs were recorded in term of the National *Cancer* Institute-Common Terminology Criteria for Adverse Events (NCI-CTCAE) (version 5.03).

### Immunogenicity Evaluation

5.0 ml blood sample were collected in vacuum tubes containing separation gel at 0 h (pre-dose) and day15 (336 h), 43 (1,008 h) and 85 (2016 h) to detect the incidence and titer of anti-drug antibody (ADAs). The ADA-positive samples were further examined for the presence of neutralizing antibodies (NAbs).

### Sample Size Estimation

The maximum coefficient of variation (CV%) for C_max_ was approximately 25% for bevacizumab in healthy subjects (Hettema et al., 2007). In our study, geometric mean ratio (GMR) was set to be 95–105% to achieve 90% power (1-β) at the 5% nominal level (*α* = 5%). If HOT-1010 and bevacizumab (Avastin®) were assumed to be bioequivalence, 90% Confidence Interval (CI) for the ratio should be within 80–125%, the initial estimated sample size was 37 in each group, calculated by SAS software (version 9.4). Considering the 10% drop-out rate and random grouping, the final sample size was 84 (42 male subjects per group).

### Statistical Analysis

PK analysis was performed by a non-compartmental method using Phoenix WinNonlin software (version 8.2). C_max_ and T_max_ were directly obtained from serum concentration data. AUC_0-t_ was calculated using the linear/log trapezoidal method. The elimination rate constant (λz) was estimated using linear least-squares regression analysis of the serum concentration-time data obtained during the terminal log-linear phase. T_1/2_ was calculated as 0.693/λz. AUC_0-∞_ was calculated as AUC_0-∞_ = AUC_0-t_ + Ct/λz, where Ct was the last detectable concentration. The criteria of PK similarity between HOT-1010 and Avastin® was met, when the 90% CIs for GMRs of C_max_, AUC_0-t_ and AUC_0-∞_ fall within the range of 80.00–125.00%.

The Full Analysis Set (FAS) and Safety Set (SS) included all randomized subjects who received at least one dose of trial medication. The Pharmacokinetic Set (PKS) included all subjects who completed the study without any major protocol deviations impacting PK profile and had measurable concentration. Descriptive statistics were calculated for PK parameters and demographical data. Variance analysis and *t*-test for normally distributed data and Wilcoxon rank test for non-normally distributed data were used for data analysis. All statistical analyzes were performed with SAS software (version 9.4). *p* < 0.05 was considered statistically significant.

## Results

### Demographic Data of Subjects

**FIGURE 1:**
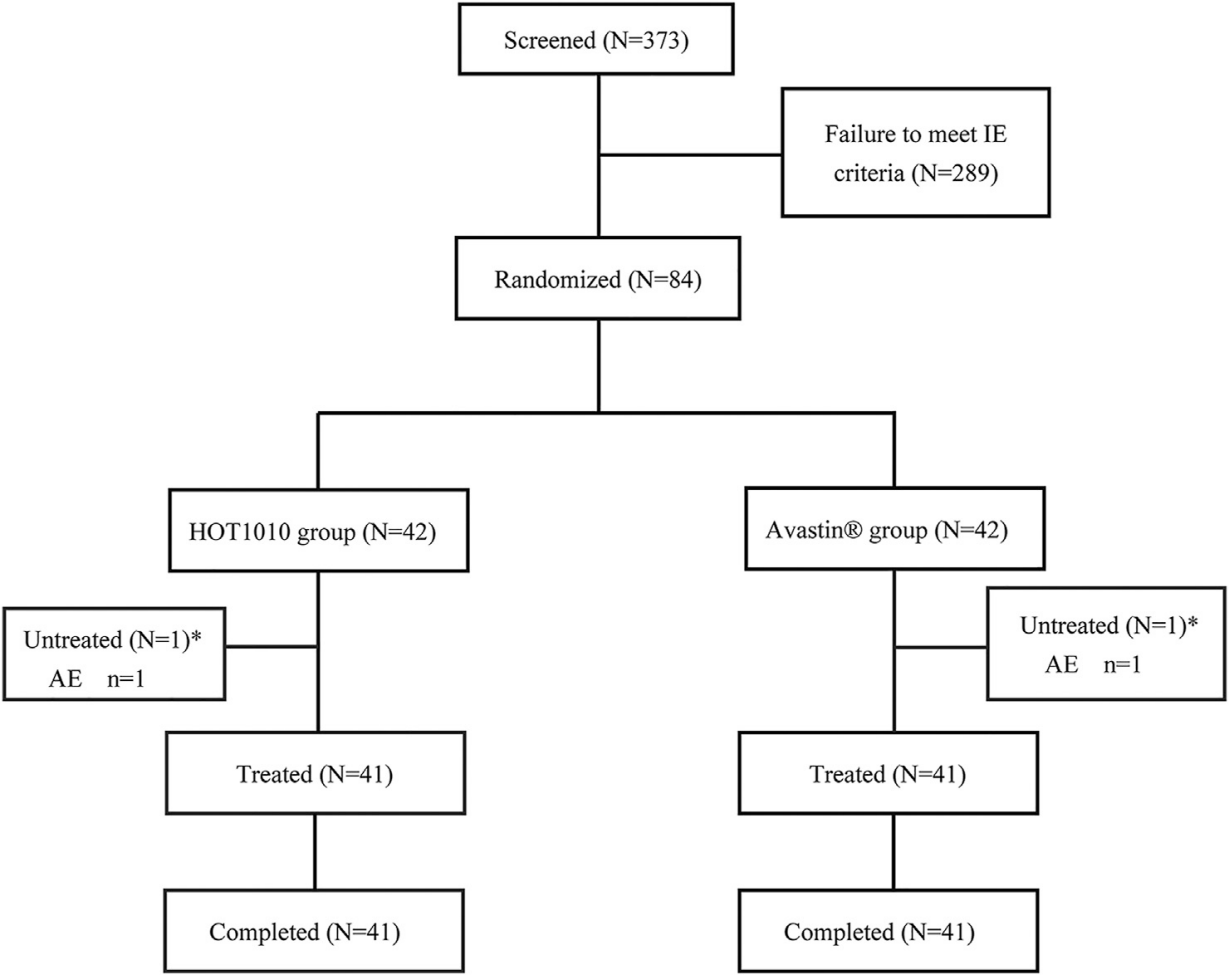
Subjects dispositions for HOT-1010 group vs. bevacizumab group. *Mild abnormality in ECG was found before dosing. IE: inclusion/exclusion.

In the screening period, 373 male subjects were enrolled and signed the informed consent form. 289 subjects failed to meet the enrollment criteria, 84 subjects were enrolled. Before the treatment, the block randomization method was used to randomly assign the subjects into HOT-1010 group (*n* = 42) or Avastin® group (*n* = 42) in a 1:1 ratio according to the random table. Eventually, 82 subjects received a single dose of the investigational drug (HOT-1010, n = 41; Avastin®, *n* = 41) and completed the study. 1 subject in each group experienced a non-drug related AE which was the mild abnormality in ECG, and dropped out before dosing (Figure 1). 82 subjects were all included in the FAS, PKS and SS. Demographics and other baseline characteristics were well matched between HOT-1010 group and Avastin® group (Table 1). Additionally, there were no clinically significant abnormalities on baseline values of vital signs, ECG, physical examination and laboratory tests.

**TABLE 1:** Demographics and baseline characteristics of Chinese healthy male subjects.

Parameter	HOT-1010 group (*N* = 41)	Avastin® group (*N* = 41)	Total (*N* = 82)
Ethnicity (Han/other)	40/1	40/1	80/2
Age (years)	28.3 ± 7.03	29.3 ± 7.59	28.8 ± 7.29
Height (cm)	169.51 ± 6.100	169.31 ± 6.339	169.41 ± 6.183
Weight (kg)	65.13 ± 5.570	64.13 ± 6.125	64.63 ± 5.840
BMI (kg/m^2^)	22.68 ± 1.726	22.36 ± 1.671	22.52 ± 1.695

BMI, body mass index; Data are presented in mean ± SD unless otherwise indicated.

### Pharmacokinetic and Statistical Analyses

**FIGURE 2:**
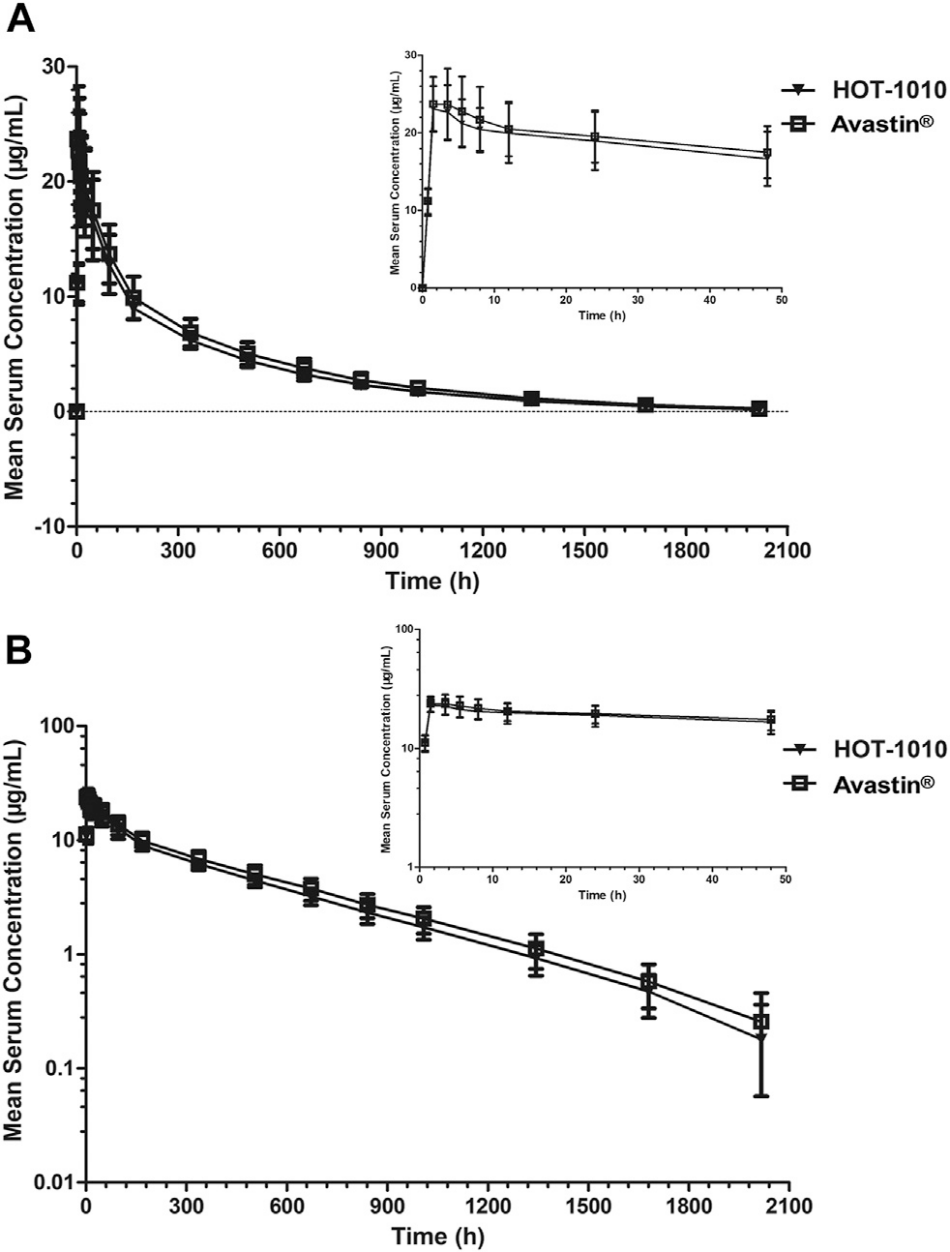
Mean serum concentrations ±SD vs. time profiles following intravenous infusion of 1 mg/kg HOT-1010 (*n* = 41) or bevacizumab (Avastin®) (*n* = 41) in Chinese healthy male subjects on linear **(A)** and semilogarithmic scales **(B)**.

The primary PK parameters of between HOT-1010 group and Avastin® group were listed **in** Table 2, C_max_ were 24.3 ± 3.70 and 25.1 ± 4.96 μg/ml, AUC_0–t_ were 6,770.77 ± 883.299 and 7,561.86 ± 1,329.929 h μg/mL,AUC_0–∞_ were 6,935.88 ± 914.949 and 7,755.94 ± 1,383.623 h μg/mL, respectively. There were no significant differences (*p* > 0.05) in primary PK parameters. Figure 2 showed the mean serum concentration-time curves after intravenous infusion of 1 mg/kg HOT-1010 or Avastin® in Chinese healthy male subjects. The statistical comparisons of PK parameters were summarized in Table 3. The mean ratios of C_max_, AUC_0-t_ and AUC_0-∞_ for HOT-1010/Avastin® were 97.54, 90.15 and 90.05%, respectively. The maximum inter-subject CV% was less than 20%. The 90% CIs for GMRs of C_max_, AUC_0-t_ and AUC_0-∞_ were in the range of 91.81–103.64%, 85.19–95.39% and 85.04–95.36%, which were all within 80.00–125.00%.

**TABLE 2:** The primary pharmacokinetic parameters of bevacizumab between HOT-1010 group and Avastin® group.

Parameter	HOT-1010 group (*N* = 41) Avastin® group (*N* = 41)	
C_max_ (μg/ml)	24.3 ± 3.70	25.1 ± 4.96
AUC_0–t_ (h•μg/mL)	6,770.77 ± 883.299	7,561.86 ± 1,329.929
AUC_0–∞_ (h•μg/mL)	6,935.88 ± 914.949	7,755.94 ± 1,383.623
T_max_ (h)^a^	3.48 (1.50, 47.95)	1.50 (1.50, 47.93)
t_1/2_ (h)	351.61 ± 50.010	359.44 ± 52.023
Vd (ml/kg)	73.67 ± 9.311	67.72 ± 8.070
CL (ml/h/kg)	0.15 ± 0.02	0.13 ± 0.02

a Median (minimum, maximum).

**TABLE 3:** Statistical comparison of primary pharmacokinetic parameters of bevacizumab.

Parameter	Geometric mean		T/R (%)	90% confidence interval	Inter-subject CV (%)
	HOT-1010 group (T)(*N* = 41)	Avastin® group (R)(*N* = 41)			
Cmax (μg/ml)	24.0	24.6	97.54	91.81–103.64	19.8
AUC_0–t_ (h•μg/mL)	6,715.6	7,449.8	90.15	85.19–95.39	17.6
AUC_0–∞_ (h•μg/mL)	6,878.0	7,637.9	90.05	85.04–95.36	17.8

### Safety Analysis

50 subjects (61.0%) experienced at least one treatment-emergent adverse event (TEAE) during the study. TEAEs were reported in 27 (65.9%) subjects in HOT-1010 group and 23 (56.1%) subjects in Avastin® group, 27 subjects and 21 subjects of which experienced TEAEs related to the investigational drug, respectively. The majority of TEAEs were mild (Grade Ⅰ) or moderate (Grade Ⅱ), Grade Ⅲ TEAEs were increased blood creatine phosphokinase (*n* = 1, 2.4%), avulsion (*n* = 1, 2.4%, unrelated to the drug) and arthralgia (*n* = 1, 2.4%) only reported in HOT-1010 group. The increased blood triglyceride was the most common TEAE found in HOT-1010 group (*n* = 7, 17.1%) and Avastin® group (*n* = 6, 14.6%), there were no TEAEs, SAEs or deaths leading to discontinuation (Table 4). Overall, 4 subjects had used a transient concomitant medication, three from HOT-1010 group (external otitis, *n* = 1, 2.4%; avulsion, *n* = 1, 2.4%; arthralgia and arthritis, *n* = 1, 2.4%) and one from Avastin® group (diarrhea, *n* = 1, 2.4%), others all recovered spontaneously. None of the subjects showed serious hypersensitivity, anaphylaxis, or injection-site reaction after intravenous infusion. All AEs had been reported to the Ethics Committee of Wuxi people’s hospital.

**TABLE 4:** Summary of treatment-emergent adverse event.

Category	HOT-1010 group (*N* = 41) n (%)	Avastin® group (*N* = 41) n (%)	Total (*N* = 82) n (%)
TEAE	27 (65.9)	23 (56.1)	50 (61.0)
*TEAE Severity*			
Grade 1	27 (65.9)	22 (53.66)	47 (57.32)
Grade 2	2 (4.88)	1 (2.44)	3 (3.66)
Grade 3	2 (4.88)	0 (0)	2 (4.9)
Grade 4 or 5	0 (0)	0 (0)	0 (0)
*TEAE Causality*			
Not related	4 (9.76)	2 (4.88)	6 (7.32)
Related	27 (65.9)	21 (51.2)	48 (58.5)
SAE	0 (0)	0 (0)	0 (0)
*TEAEs occurring in 5% of subjects in any treatment group*			
Blood triglycerides increased	7 (17.1)	6 (14.6)	13 (15.9)
Alanine aminotransferase increased	5 (12.2)	2 (4.9)	7 (8.5)
Aspartate aminotransferase increased	5 (12.2)	2 (4.9)	7 (8.5)
Hyperuricemia	5 (12.2)	2 (4.9)	7 (8.5)
Blood fibrinogen decreased	4 (9.8)	2 (4.9)	6 (7.3)
Conjugated bilirubin increased	3 (7.3)	2 (4.9)	5 (6.1)
Blood creatine phosphokinase increased	3 (7.3)	0 (0)	3 (3.7)
Proteinuria	0 (0)	4 (9.8)	4 (4.9)

HOT-1010 was safe and well-tolerated in Chinese healthy male subjects during the study and demonstrated the safety profiles were comparable to bevacizumab.

### Immunogenicity Evaluations

82 subjects were tested negative for ADA before (0 h) and after intravenous infusion on the day 15, 43 and 85, respectively.

## Discussion

None of the biosimilars of bevacizumab so far has been marketed in China. It is the first clinical trial of HOT-1010 conducted in Chinese healthy male subjects to approve its biosimilarity to bevacizumab. The results demonstrated that HOT-1010 has similar PK profiles to Avastin® when administered in healthy male subjects. The 90% CIs for GMRs of C_max_, AUC_0-t_ and AUC_0-∞_ were within the bioequivalence acceptance range of 80–125.00%.

This study was designed as a randomized, double-blind, parallel study in healthy subjects. The blinded manner could eliminate assessment bias, especially safety assessment and the potential influence of immunogenicity on PK parameters, so drug administration and safety assessment were all conducted in a blinded manner. The parallel design was considered more appropriate than cross-over due to the long half-life of bevacizumab (approximately 20 days) (Lu et al., 2008), blood samples were collected last for 85 days to characterize PK profiles of bevacizumab adequately.

Healthy subjects could be the most sensitive population for assessing the similarity of PK. The tumor patients had complicated disease complications and concomitant medications, which possibly altered PK profiles of HOT-1010 and bevacizumab. In addition, gender difference also influenced PK profiles of bevacizumab, which exhibited about 26% higher clearance in males than in females, and increased the risk of ovarian failure and possibly impaired female fertility (Lu et al., 2008). The enrollment of healthy male subjects ensure the study population's homogeneity and improve the sensitivity of detecting potential PK differences between two groups (NMPA CHINA, 2020).

The linear PK profile of bevacizumab was found over the dose range of 1–10 mg/kg in tumor patients (Lu et al., 2008). Therapeutic doses of bevacizumab were 5–15 mg/kg every 2–3 weeks (Genentech Inc, 2015; Socinski et al., 2015). In order to reduce drug exposure in healthy subjects, the minimal dose of bevacizumab (1 mg/kg) was chosen in this study, which was obviously less than the recommended clinical dose for cancer treatment (Genentech Inc, 2015). Therefore, we confirmed that 1 mg/kg bevacizumab minimized the potential risk to healthy subjects and obtained meaningful PK results considering the LLOQ of the analytical method.

In our study, HOT-1010 showed similar PK profiles to Avastin®. There was almost no difference in primary PK parameters between HOT-1010 and Avastin®. The mean t_1/2_ of HOT-1010 and Avastin® was 14.6 d and 14.9 days, which was consistent with the results of other bevacizumab biosimilars (13.1–19.3 days) in Chinese, Indian, Caucasian and Korean healthy male subject studies (Knight et al., 2016; Zhang et al., 2018; Wang et al., 2019; Liu et al., 2020; Shin et al., 2020; Singh et al., 2020). The median T_max_ of HOT-1010 (3.48 h) was similar to that of bevacizumab and other bevacizumab biosimilars (2.5–4.5 h) when intravenous dose ranged from 1 to 3 mg/kg (Zhang et al., 2018; Wang et al., 2019; Liu et al., 2020; Shin et al., 2020; Singh et al., 2020).

HOT-1010 was well-tolerated and no safety concerns were identified, and safety profiles were similar between HOT-1010 group and Avastin® group. The most common TEAEs (more than 5% incidence) included increased blood triglycerides, increased alanine aminotransferase, increased aspartate aminotransferase, hyperuricemia, decreased blood fibrinogen, increased conjugated bilirubin, increased blood creatine phosphokinase and proteinuria. TEAEs reported in this study were almost expected, based on previous studies in healthy subjects and tumor patients (Genentech Inc, 2015; Liu et al., 2020; Wang et al., 2019). All subjects with TEAEs had recovered. There were no serious AEs and deaths during the study. The incidence of increased blood triglycerides was the highest in HOT-1010 group and Avastin® group (17.1 and 14.6%). The case of bevacizumab-associated hyperlipoproteinemia has also been reported in a patient with advanced breast cancer (Joerger et al., 2011). It has been found that there was some potential link between angiogenesis and adipocyte activity, activated adipocytes could produce multiple angiogenic factors (VEGF, leptin, angiopoietins, HGF and so on) (Cao, 2007). Anti-angiogenic drugs might have some anti-atherogenic activities, a hypothetical release of cholesterol and triglycerides from endothelial sources might cause the increase of serum lipids. Disturbances of lipid metabolism after administration might be another explanation (Joerger et al., 2011). Arthralgia and arthritis were two noteworthy AEs found in a healthy subject who was only given a single dose of HOT-1010. In previous studies on the safety of bevacizumab, arthralgia has been found in some cancer patients who were chronically administered bevacizumab with or without chemotherapy. However, it was still hard to confirm the causality between bevacizumab and arthralgia, which was influenced by various factors (Genentech Inc, 2015; Vauléon et al., 2021). Arthralgia and arthritis were also reported in the hereditary hemorrhagic telangiectasia (HHT) patients treated by intravenous bevacizumab for a long time (Guilhem et al., 2017). It is hypothesized that hypoxia may be an initiating driver of inflammatory processes in the arthritic joint due to the anti-VEGF effect (Ng et al., 2010; Konisti et al., 2012). Although the exact mechanisms are not clear yet, these AEs of HOT-1010 should be paid more attention in the future study. Additionally, 82 subjects were found to be negative for ADA test, other studies also showed the relatively low immunogenicity of bevacizumab (Knight et al., 2016; Zhang et al., 2018; Wang et al., 2019; Liu et al., 2020; Shin et al., 2020; Singh et al., 2020), the comparison of immunogenicity still needs to be further investigated with larger sample size.

Currently, a multicenter, randomized, double-blind, parallel-group, phase Ⅲ clinical trial in patients with advanced, metastatic or recurrent non-squamous NSCLC is underway to evaluate the efficacy, safety and immunogenicity of HOT-1010 vs. bevacizumab (Avastin®) in combination with paclitaxel and carboplatin.

## Conclusion

The present study demonstrated the PK similarity of HOT-1010 with bevacizumab (Avastin®). Intravenous infusion of 1 mg/kg HOT-1010 was safe and well-tolerated in Chinese healthy male subjects. These findings support the clinical development of HOT-1010 as the biosimilar of bevacizumab.

## Data Availability

The original contributions presented in the study are included in the article/Supplementary Material, further inquiries can be directed to the corresponding author.
